# Iron Homeostasis and Inflammatory Status in Mice Deficient for the Cystic Fibrosis Transmembrane Regulator

**DOI:** 10.1371/journal.pone.0145685

**Published:** 2015-12-28

**Authors:** Jean-Christophe Deschemin, Sarah Allouche, Franck Brouillard, Sophie Vaulont

**Affiliations:** 1 INSERM, U1016, Institut Cochin, Paris, France; 2 CNRS, UMR8104, Paris, France; 3 Université Paris Descartes, Sorbonne Paris Cité, Paris, France; 4 Laboratory of Excellence GR-Ex, Paris, France; 5 Institut de Chimie des Substances Naturelles, UPR2301 CNRS, Gif-sur-Yvette, France; CINVESTAV-IPN, MEXICO

## Abstract

**Background:**

Cystic Fibrosis (CF) is a frequent and lethal autosomal recessive disease caused by mutations in the gene encoding the Cystic Fibrosis Transmembrane Conductance Regulator (CFTR). Patients with CF suffer from chronic infections and severe inflammation, which lead to progressive pulmonary and gut diseases. Recently, an expanding body of evidence has suggested that iron homeostasis was abnormal in CF with, in particular, systemic iron deficiency and iron sequestration in the epithelium airway. The molecular mechanisms responsible for iron dysregulation and the relationship with inflammation in CF are unknown.

**Methods and Results:**

We assessed the impact of CFTR deficiency on systemic and tissue iron homeostasis as well as inflammation in wildtype and CFTR knockout (KO) mice. First, in contrast to the systemic and intestinal inflammation we observed in the CFTR KO mice, we reported the absence of lung phenotype with regards to both inflammation and iron status. Second, we showed a significant decrease of plasma ferritin levels in the KO mice, as in CF patients, likely caused by a decrease in spleen ferritin levels. However, we measured unchanged plasma iron levels in the KO mice that may be explained by increased intestinal iron absorption.

**Conclusion:**

These results indicate that in CF, the lung do not predominantly contributes to the systemic ferritin deficiency and we propose the spleen as the major organ responsible for hypoferritinemia in the KO mouse. These results should provide a better understanding of iron dysregulation in CF patients where treating or not iron deficiency remains a challenging question.

## Introduction

Despite the clear link between abnormal ion transport by loss-of function mutations in CFTR and CF, the pathogenesis of the disease is still poorly understood. It involves multiple organs, but the lung and the digestive diseases are the major causes of morbi-mortality [1].

The CF pulmonary disease is initiated in airways, that become obstructed after birth with mucus plugs due to impaired epithelial chloride (Cl-) ion channeling, sodium (Na+)-dependent water transport, and decreased clearance of mucus secretion [2]. These thick mucus plaques act as a nidus for persistent bacterial infection (*P*. *aeruginosa* -PA- remaining the critical determinant of pulmonary pathology), resulting in increased airway inflammation, oxidative stress and finally to respiratory failure [3]. Importantly, it was reported that even in the absence of clinically apparent infection, inflammation is often present in CF airways, as evidenced by neutrophil accumulation and excessive concentrations of interleukin (Il) 8 and free proteases [4]. The gastrointestinal (GI) disease is the first hallmark of CF in a significant number of affected newborns that present with obstructive meconium ileus, and remains a major cause of morbidity throughout life. As in airways, mucus secretions in the GI tract are more viscous and dehydrated, also as a result of abnormal fluid flow. The CF intestine also exhibits an inflammatory status with up-regulation of components of the innate immune system [5].

An expanding body of evidence suggests that iron homeostasis is abnormal in CF. In one hand, systemic iron deficiency (ID), as defined by the criteria of the World Health Organization i.e. ferritin less that 15 μg/L, is common in CF [6]. Its clinical significance remains however uncertain. It is usually attributed to a combination of factors including chronic inflammation, poor dietary intake, and GI blood loss. In addition, although a direct effect of PA infection on ID has not been clearly established, the ability of PA to obtain extracellular iron from host tissues for growth and enhancement of virulence suggests that this pathogen may play a role in depleting body iron stores. In this line, Moreau-Marquis *et al*. demonstrated that human bronchial epithelial cells from CF patients accumulate and release more iron in the apical medium in which PA forms biofilms than control cells, and that this increased iron facilitates biofilm formation [7]. More recently, downregulation of heme oxygenase-1 (HO-1) was proposed to be responsible for iron accumulation in human CF epithelial cells [8]. On the second hand, abnormal airway iron levels have been described by a number of laboratories in CF patients [9–11]. In these studies, increase iron in the airway, in the sputum and in the macrophages (as seen in explanted lung by Ghio *et al*. [11]) were described. In fact, this sequestration of iron in the airways has also been proposed to contribute to the iron deficiency in CF patients.

Maintenance of iron homeostasis on the level of whole organism is primarily mediated by the iron-regulatory hormone hepcidin [12]. This hormone is acting to ensure adequate supply of iron to erythroid precursors, the main iron consumers, but also to all other cells for vital metabolic processes. Hepcidin is produced primarily in hepatocytes and controls plasma and tissue iron levels by regulating the delivery of iron to plasma through the iron exporting protein ferroportin, the sole known cellular iron exporter in vertebrates. Ferroportin is mainly expressed in cells processing large amounts of iron, in particular the enterocytes of the duodenum, involved in dietary iron absorption, and the macrophages of the spleen that recycle iron from senescent red blood cell (RBC) through a complex erythrophagocytosis process. Hepcidin acts by binding to ferroportin, triggering its ubiquitination and degradation into the lysosome, decreasing in turn iron delivery and leading thus to hypoferremia [13]. Recent report also suggest that in the gut, hepcidin may reduce iron absorption by decreasing the expression of the apical iron transporter Divalent metal-ion transporter 1 (DMT1), although the molecular mechanisms have yet to be established [14].

At the cellular level, iron homeostasis is orchestrated at a post transcriptional level by the Iron Regulatory Proteins (IRP1 and 2) binding to RNA stem-loop structures called iron-responsive elements (IREs) in transcripts encoding proteins involved in iron uptake (DMT1 and transferrin receptor 1, TfR1), storage (ferritin) and export (ferroportin) [15]. IRP activity is regulated by intracellular iron levels (and other iron-independent mechanisms) to prevent iron deficiency and impairment of vital cellular functions, and, conversely, cellular iron overload, which leads to the generation of toxic radicals via an iron-catalyzed Fenton reaction.

So far, the molecular mechanisms responsible for dysregulation of iron homeostasis in CF are unknown and provide the rationale of this study where we looked at the impact of CFTR deficiency on systemic iron metabolism in CFTR knockout (KO) mice. CF being also characterized by excessive host inflammatory response [16] and iron homeostasis being intimately tied to the inflammatory [17] response, we also looked at the inflammatory status of the CFTR KO mice.

## Material and Methods

### Animals

Mice were cared for in accordance with the European convention for the protection of laboratory animals. Animal studies received approval from the Regional Ethics Committee for Animal Experimentation of University Paris Descartes. Mice lacking CFTR expression (established by gene targeting: cftrtm1Unc, CFTR KO mice, [18]) were obtained from the Animal Core Facility at Centre de Distribution, Typage and Archivage animal (Service CNRS, Orléans, France). These mice were maintained on a mixed genetic background (129/BC-C57BI/6). CFTR KO mice or their wild-type (WT) littermate controls were provided after weaning, a commercial osmotic laxative (Movicol^®^; Norgine, Middlesex, UK) in the drinking water to prevent intestinal obstruction. This treatment allows increasing the survival of the CFTR KO mice. All mice used were males between 20 and 24 weeks of age. Results are presented for n = 4 animals per group. Hematologic parameters were measured on a MS9-5V apparatus (Melet Schloesing Laboratories).

### Iron and plasma measurements

Plasma iron, transferrin and ferritin content were measured on an Olympus AU400 automat. Transferrin saturation was calculated with the formula: (plasma iron x 100) / (plasma transferrin x 25). Tissue iron was determined by acid digestion of samples followed by determination of the iron content by a colorimetric assay on the Olympus AU400 automat. For iron staining, tissues were fixed in 4% formaldehyde and embedded in paraffin. Slides were stained with Perls’ Prussian blue and nuclear fast red counter stain using standard procedures.

### Reverse transcription and real-time quantitative PCR

RNA extraction, reverse transcription and quantitative PCR were previously described [19, 20]. All samples were normalized to the threshold cycle value for cyclophilin-A.

### Western blot

Membrane and cytosolic fractions were prepared as described [21]. Briefly, proximal duodenum tissue was incubated with 1.5 mM EDTA in PBS supplemented with PMSF and protease inhibitors, for 45 min at 4°C, with shaking. The remaining duodenal muscle was removed and the detached enterocytes were collected by centrifugation. The cells were treated with lysis buffer (0.25 M sucrose, 0.03 M L-Histidine, pH = 7.2, 500 μM PMSF and protease inhibitors) for 30 min. The samples were then centrifuged, the supernatant was removed and spun at 41,000 rpm for 1 hour to obtain the cytosolic fraction (supernatant) and the membrane fraction (pellet) was resuspended in 10 mM Tris HCl pH 7–10 mM EDTA—100 mM NaCl.

Western blot (WB) experiments were performed as previously described [21]. We used the following antibodies: anti-Dcytb (DCYTB11-A from Alpha Diagnostic; dilution 1/500); anti-DMT1 ([22], dilution 1/500); anti-ferroportin (MTP11-A from Alpha Diagnostic; dilution 1/500); anti-L-ferritin (SAB2500431 from Sigma-Aldrich; dilution 1/500) and anti-β-actin (Ascites fluid A5316 from Sigma-Aldrich, dilution 1/6000). The secondary antibodies used were anti-rabbit, anti-goat or anti-mouse (Calbiochem, dilution 1/6000), as appropriate. Briefly, samples were analyzed by SDS-PAGE and transferred onto nitrocellulose membrane in Tris/glycine buffer. Saturation was performed with 10 mM Tris buffer (pH 7.5), 150 mM NaCl, 0.05% Tween 20 (TBST), and 5% (w/v) non-fat milk powder. All primary antibodies were incubated with the membrane overnight, at 4°C, on a rocking platform. Membranes were washed in TBST and probed with the appropriate secondary antibody in TBST + 5% (w/v) non-fat milk powder, for one hour, at room temperature, on a rocking platform. Membranes were thoroughly washed and chemiluminescence was detected with Supersignal West Pico, Supersignal West Dura substrates (Thermo). Proteins were visualized with Image Quant Las4000 mini (GE Healthcare).

Western Blot with membrane extracts were performed with 10 μg/lane for the duodenum and 40 μg for the spleen. For cytosol extracts, 30 μg/lane were loaded for the duodedum and the macrophages (EP experiments) and 40 μg for the spleen.

Densitometry of the immunoblots was performed using ImageJ software. All analyses were performed by means of GraphPadPrism 4 software.

### Bronchoalveolar lavage

For BAL cell harvest, mice received ketamine (90 mg/kg) and xylazine (10 mg/kg) i.p. Briefly, the trachea was cannulated and the lungs were lavaged with a total of 3 ml of PBS in 0.5-ml aliquots. Lavage aliquots for each animal were pooled, and the cell pellet collected by centrifugation. The Perl’s prussian blue staining was performed on cytospin preparation.

### Red blood cells ageing and phagocytosis assay

Assays were performed as described [23]. Briefly, red blood cells (RBC) were treated with 2.5mM calcium and 0.5 μM of Ca2+ ionophore A23187 at 30°C for 18h. Untreated (stored at 4°C) or aged RBCs were then recovered by centrifugation and counted before use. Bone-marrow derived macrophages were incubated with normal or aged RBCs (10^7^cells/ml/well in 24 well-plate) for 1 hour at 37°C and then treated for 5 min in hypotonic solution to lyse non-ingested RBCs. After phagocytosis, cell viability was assessed by Trypan blue staining. For CFTR inhibition, bone-marow derived macrophages were treated for 2 days with CFTR inhibitor (CFTR (inh)-172, 10^-5^M) in medium renewed 3 times and 1 h before the EP. Cells were lysed 6 hours after EP to measure ferritin levels.

### Statistical analysis

Statistical analysis was performed using non-parametric Mann-Whitney tests. P values less than 0.05 were considered statistically significant.

## Results and Discussion

### Inflammatory status in CFTR KO animals

**Fig 1:**
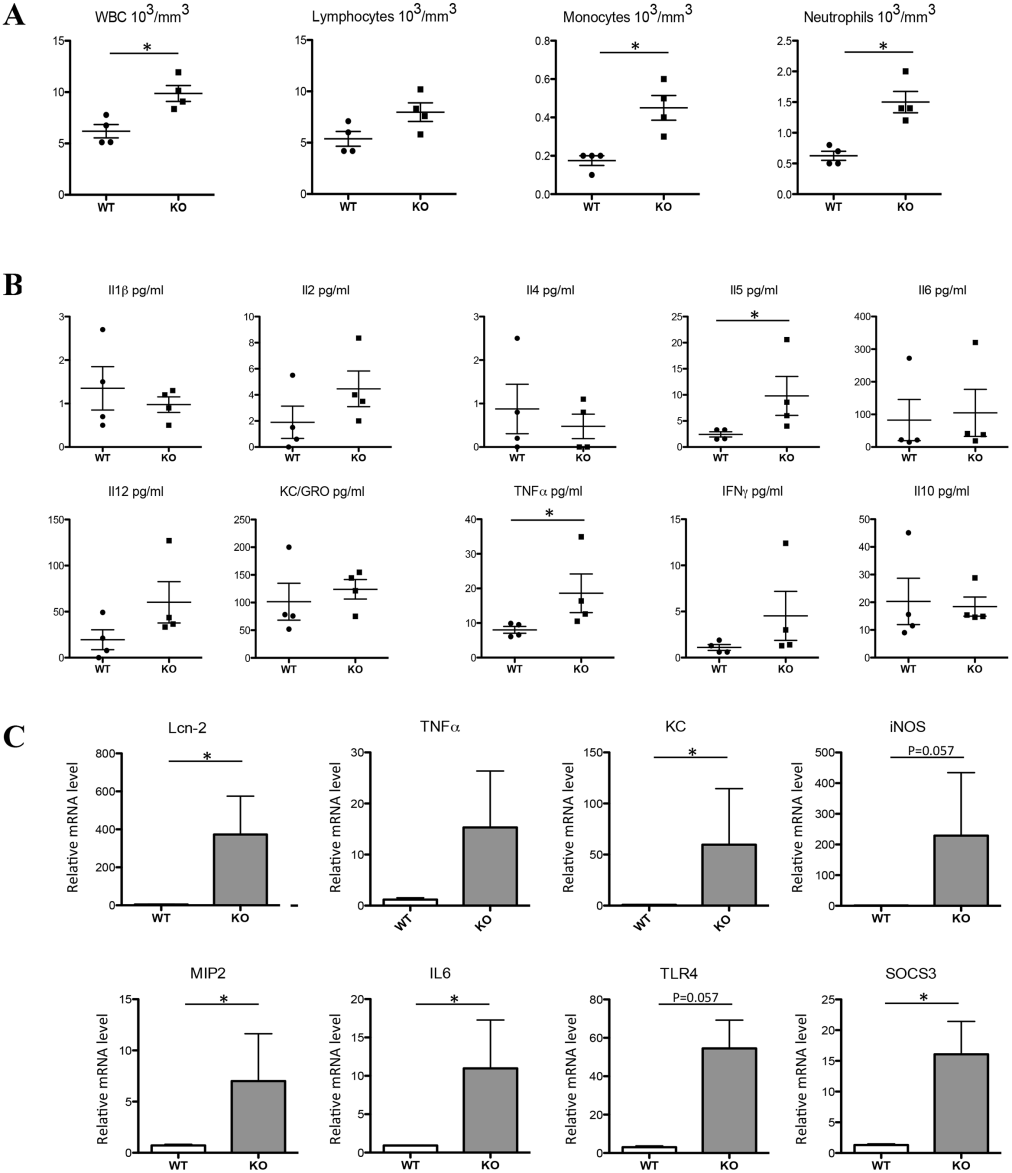
Inflammatory status of the CFTR KO mice. WBC (white blood cells), lymphocytes, monocytes and neutrophils were measured in the blood of WT and CFTR KO mice (A). Cytokines levels were measured by the V-PLEX Proinflammatory Panel1 (mouse) Kit in the plasma of WT and CFTR KO mice according to the manufacturer’s instruction (B). Duodenal mRNA levels relative to cyclophilin-A expression were assessed by real-time PCR for the indicated genes (C). Data are presented as mean ± SEM. * P<0.05.

As shown in Fig 1A, total white blood cell concentration was significantly increased in CFTR KO mice, as well as monocytes and neutrophils, reflecting a potential inflammatory status of these animals. At the systemic level, a mild inflammation status was also noted. Indeed, cytokine expression profile in the blood of WT and mutant mice, as determined by cytoplex analysis, showed significant increase in the KO mice of one of the critical cytokine, tumor necrosis factor alpha (TNFα), as well as the Il5 lymphokine and a trend for three others interleukines, Il2, Il12 and interferon gamma (IFNγ), all altered in various chronic intestinal inflammatory diseases [24–27]. No difference was observed between genotype for the other markers (Fig 1B).

To get further information on this inflammatory status, several inflammatory markers were measured by q-PCR in the intestine and the lung, the two epithelia that are largely affected in the human disease. Worth mentioning however, in animal models, incomplete injury penetrance was reported, particularly in mice, depending, among others on the age and the genetic background of the animals [16]. In our study (i.e. relatively aged mice of 20–24 week-old bred on a mixed genetic background), intestine was clearly affected by CFTR deficiency (although with a great interindividual variation likely due to the mixed genetic background), with an increase of the mRNA levels of Lipocalin 2 (Lcn-2, also known in human as NGAL, neutrophil gelatinase-associated lipocalin), TNFα, KC (also known as CXCL1), inducible nitric oxide synthase (iNOS), MIP2 (also known as CXCL2), Il-6, Toll-like receptor 4 (TLR4) (Fig 1C). The significant increased expression of the anti-inflammatory SOCS3 (Suppressor of Cytokine Signalling 3 gene) marker suggests that it may act to limit intestinal inflammation in this model. The most dramatic up-regulation was seen for Lcn-2, a small secreted protein with various activity including the control of iron availability (by binding to bacterial iron siderophores) and stimulation of inflammation. Interestingly, an increase of Lcn-2 was demonstrated in various murine models of colitis and human inflammatory bowel disease (IBD) [28]. To the best of the author’s knowledge, the link between Lcn-2 and CFTR pathogenesis has not been made and whether Lcn-2 could constitute a biomarker of intestinal inflammation, as in IBD, merits investigation.

In sharp contrast to the intestine, we found no change in the inflammatory markers in the lung of the CFTR KO mice (data not shown) nor in the mucins mRNA levels, mucins being important components of the secretory mucus that is essential in the protection and maintenance of homeostasis of epithelial lumen [29]. This result clearly suggests that the lack of CFTR in the lung of the KO mice is not sufficient for the setting of inflammation and mucus hyperproduction that typify CF [30], and that additional inflammatory signals and bacterial byproducts are probably required.

### Hematological parameters, iron indices and liver response in CFTR KO animals

**Fig 2:**
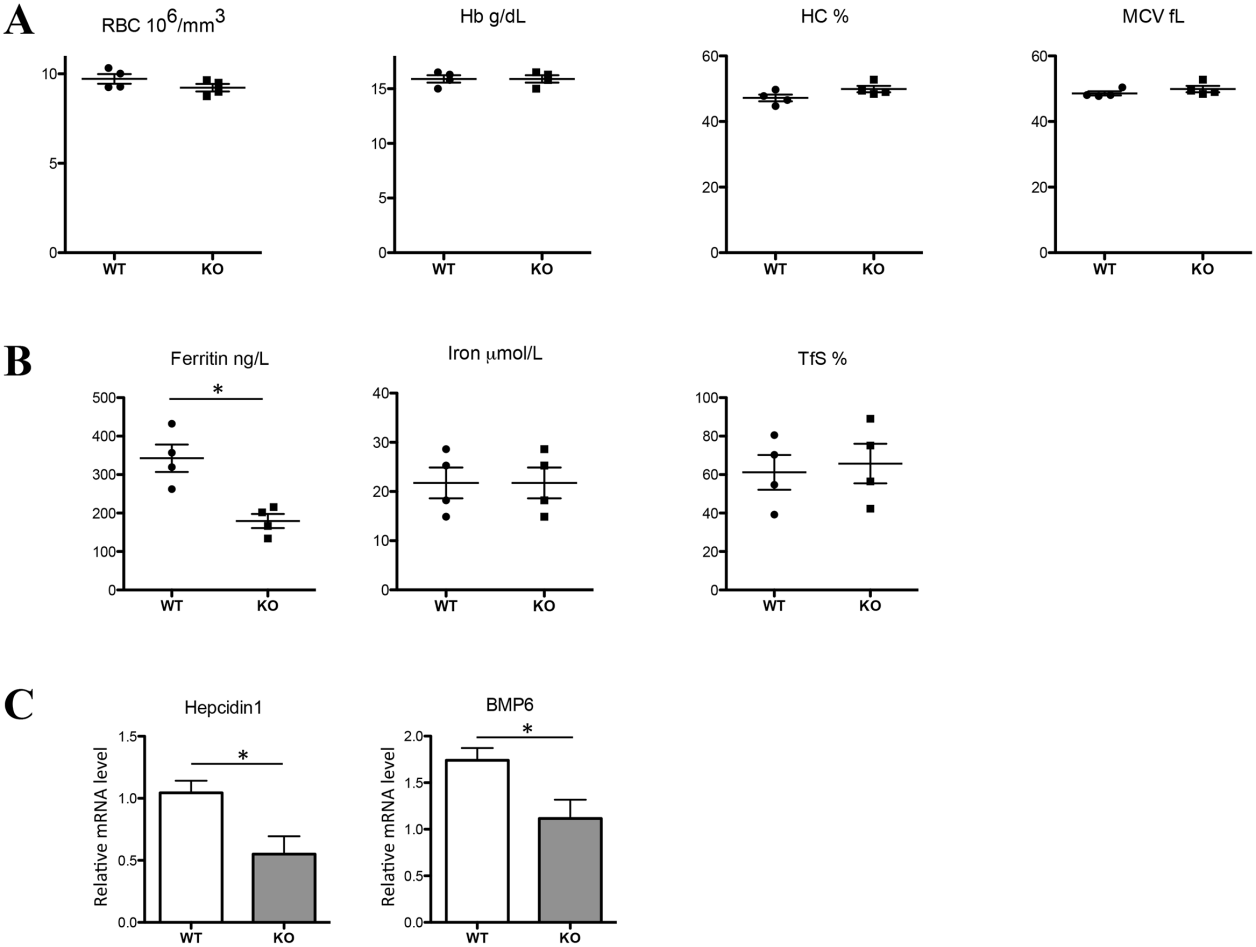
Red cells, iron indices, and iron-related genes in CFTR KO mice. Hematological and iron parameters were analyzed in WT and CFTR KO mice: RBC (red blood cells), Hb (hemoglobin), HC (hematocrit) and MCV (mean corpuscular volume) (A); plasma iron, TfS, (transferrin saturation) and ferritin (B). Liver hepcidin1 and BMP6 mRNA levels relative to cyclophilin-A were analyzed by real-time PCR (C). Data are presented as mean ± SEM. * P<0.05.

As shown in Fig 2A, none of the red cell indices were affected by CFTR deficiency. Confirming the absence of apparent dyserythropoiesis (normal RBC count and hemoglobin level), we found similar erythropoietin (EPO) mRNA levels in the kidney of WT and CFTR KO mice (not shown). Concerning the plasma iron parameters, we found a significant 35% decreased of plasma ferritin with, however, no change in iron levels nor in transferrin saturation (Fig 2B). The decrease of circulating ferritin is a well-recognized marker of iron deficiency. The liver is able to sense this iron deficiency by adjusting the synthesis of the iron regulatory hormone hepcidin to satisfy in turn the body iron demand. In the CFTR KO mice, hepcidin gene expression was decreased by almost two-fold (Fig 2C). Circulating ferritin-induced hepcidin regulation was proposed by Feng *et al*. to be mediated by the level of BMP6 [31]. Accordingly, we found a significant decrease of BMP6 mRNA levels in the liver of CFTR KO mice that could thus account for hepcidin repression (Fig 2C). Apart form these changes, no feature of iron modification was observed in the liver of the CFTR KO mice, in particular, no change in total iron, L- ferritin level, and iron content as assessed by the Perl's iron stain (Prussian blue reaction), a common and reliable stain for detecting iron (S1 Fig).

**Fig 3:**
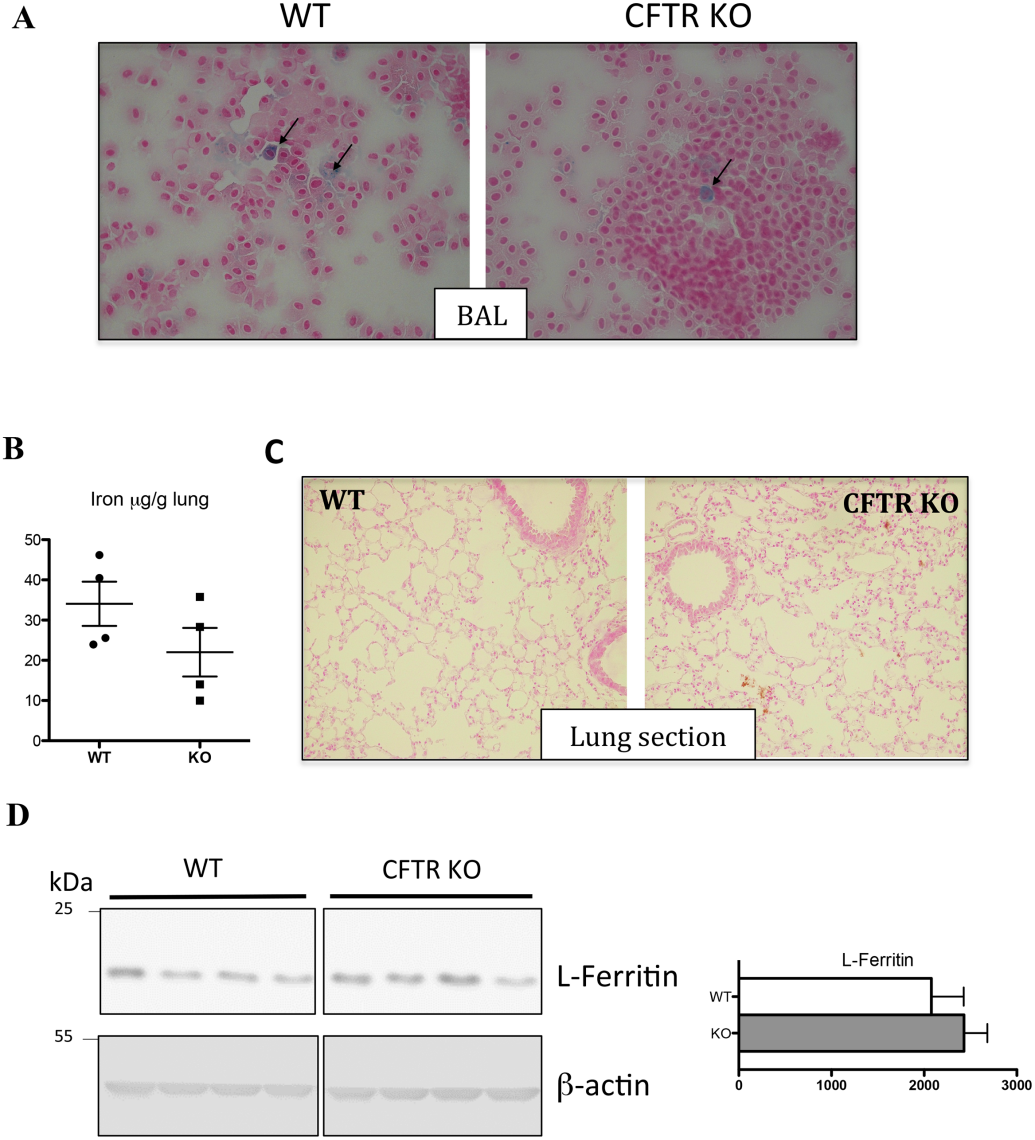
Lung iron phenotype in CFTR KO mice. Representative images of BAL (bronchoalveolar lavages) cytospin slides obtained from WT and CFTR KO mice and stained with Perl's Prussian blue. Iron loaded macrophages are indicated by arrows. Magnification X40 (A). Perls’ blue staining of lung section from WT and KO mice (B). Lung iron content (C) and L-ferritin analysis from cytosolic fractions; the right panel represents the quantification of the blots (arbitrary units) (D). Data are presented as mean ± SEM.

To determine whether the mouse model of CFTR deficiency reproduce the abnormal iron distribution in the lung of CF patients, in particular the iron accumulation in the alveolar macrophages [11], we prepared macrophages from bronchoalveolar lavage (BAL) isolated from WT and CFTR KO mice and assessed their iron content by Perl's Prussian blue staining. The BAL macrophages do not present any signs of iron overload and only a very few macrophages, in both WT and KO macrophages, presented mild iron deposit (Fig 3A). In addition, iron content in the lung was found unchanged in the KO mice (Fig 3B), no iron staining was detectable by Perl’s staining on lung section (Fig 3C), L-ferritin protein level, as assessed by WB (Fig 3D), and ferritin content in the BAL (60,56 **±** 24,76 ng/L, in WT, n = 3, vs 46,70 **±** 3,98 ng/L in KO mice, n = 3, p = 0.4) were found similar in WT and KO mice. Thus, in this mouse model, iron loading in the airway is not observed.

### Iron-related gene expression in the duodenum of CFTR KO mice

In the intestine, iron is absorbed from diet across the duodenal brush border membrane of the enterocytes by DMT1. This transport step is preceded by the reduction of iron to its ferrous (Fe2+) form thanks to the ferric reductase duodenal cytochrome b (Dcytb). At the basolateral membrane, iron export to the circulation is mediated by ferroportin. Apart form the transporters DMT1 and ferroportin, duodenal ferritin was recently shown to be also critical for regulating intestinal iron fluxes [32]. Galy *et al*. demonstrated that even with high DMT1 and ferroportin levels, iron transfer could be dramatically reduced if ferritin is hyperinduced (a phenomena referred to as “the mucosal block”) [33]. Iron absorption in the CFTR KO mice was estimated by analyzing the expression of iron-related genes in duodenal enterocytes of WT and KO mice, at both the protein and the mRNA levels.

**Fig 4:**
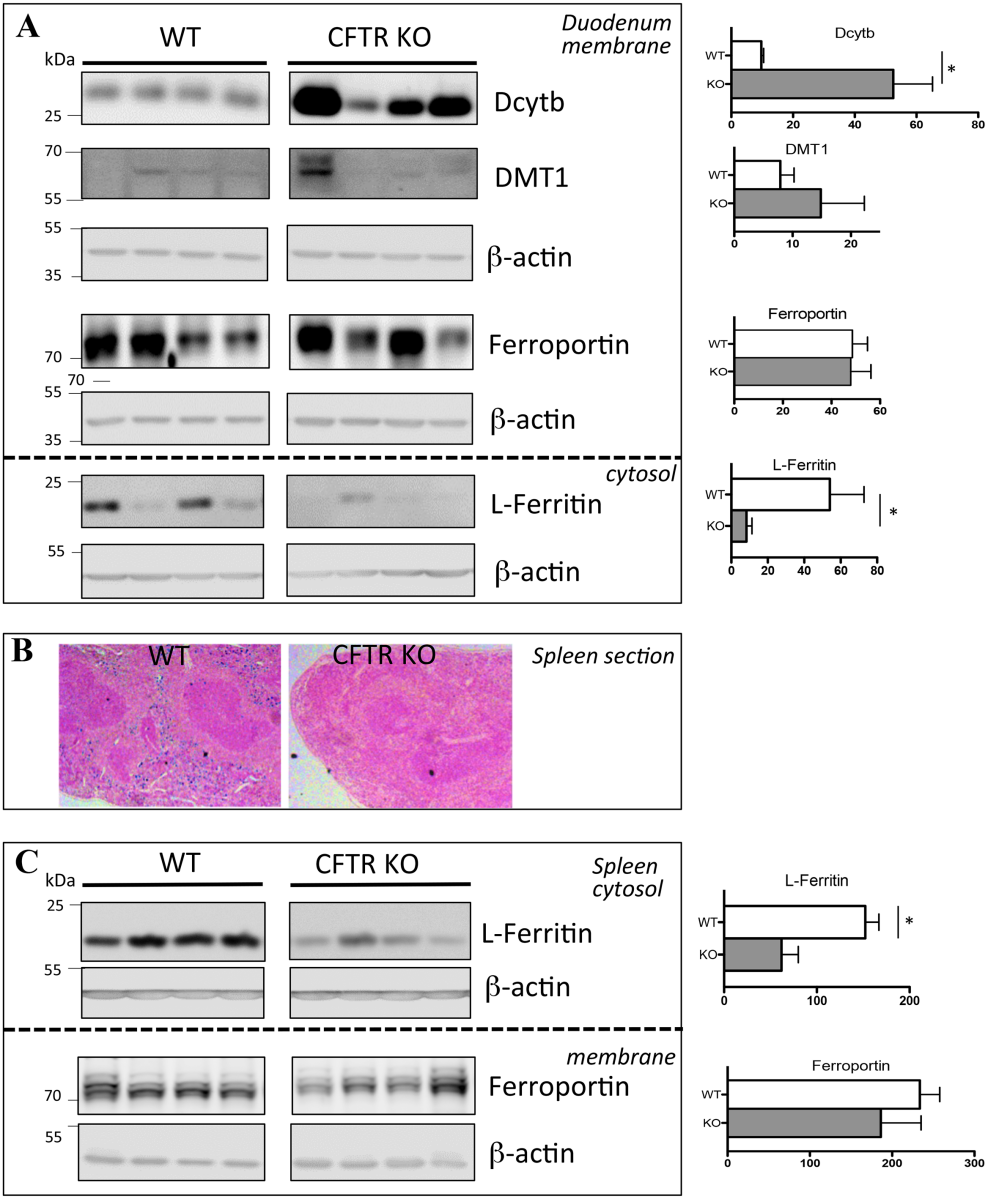
Duodenal and spleen iron-related proteins in CFTR KO mice. Duodenum (A) and spleen (B, C) were collected from WT and CFTR KO mice. Duodenum DcytB, DMT1 and ferroportin were analyzed using proteins from membrane enriched-fractions and L-ferritin from cytosolic fractions. The loading control, ß-actin, is shown (A). Perls’ blue staining of spleen section from WT and KO mice (B). Spleen ferroportin was analyzed using proteins from membrane enriched-fractions and L-ferritin from cytosolic fractions. The loading control, ß-actin, is shown. The right panel represents the quantification of the blots (arbitrary units). Data are presented as mean ± SEM. * P<0.05.

In the membrane fraction of the duodenum, we detected no consistent changes in ferroportin nor in DMT1 protein levels, although the level of this latter transporter was barely detectable (Fig 4A, top panel). This result likely indicates that the decrease of hepcidin has not reached a sufficient level to alter the level of these iron transporters. In contrast, we observed a dramatic increase in Dcytb protein expression in the CFTR KO mice as compared to WT mice. Consistent with the changes in the protein level, we measured a significant increase in Dcytb mRNA level and no change in ferroportin and DMT1 mRNA levels (S2A Fig). In view of the known down-regulation of Dcytb expression during acute [21] and chronic inflammation [34], this result suggests the existence of a dominant positive signal overriding the inhibitory effects of inflammation on Dcytb gene expression. HIF-2 has emerged these last years as a crucial factor of duodenal iron absorption that may operate independently of the systemic iron homeostatic regulators and Dcytb has been demonstrated to be highly responsive to HIF-2 [14, 35]. We found however only a trend but no significant increase of HIF-2 mRNA levels in the duodenum of CFTR KO mice (S2B Fig). Nevertheless, HIF-2 regulation in the duodenum may occur at the translational level through IRP1 [36] and therefore measurement of HIF-2 protein content is required to conclude on its potential regulatory role in the CFTR KO mice.

In the cytosol, the level of L-ferritin was importantly reduced (Fig 4A, bottom panel) with, however, no change in mRNA levels (S2B Fig), suggesting a post-transcriptional level of ferritin regulation. Thus, the increase expression of Dcytb, together with the decrease of L-ferritin (contributing to increased iron flux through reduced mucosal block), may result in enhance iron absorption that could explain the normal level of circulating iron in the CFTR KO mice. Noteworthy, the lower intraluminal intestinal pH in CF is also an important physiological variable to take into consideration since changes in duodenal pH are known to affect the driving force for iron absorption [37].

### Iron recycling by splenic macrophages in the CFTR KO mice

The majority of body iron is contained within the RBCs as a component of hemoglobin. At the end of their lifespan, senescent RBCs are phagocytosed by macrophages (essentially the macrophages of the spleen) that recycle the iron to plasma transferrin for reincorporation into new RBCs. Sections from the spleens of CFTR KO mice showed a dramatic decrease of Perls Prussian blue staining in the red pulp, featuring an iron deficient state of the mutant spleen (Fig 4B). This low staining was associated with a marked reduction in L-ferritin expression in WB of splenic extracts from CFTR KO mice (Fig 4C, top panel). The levels of the corresponding mRNAs were however unchanged (S2C Fig), suggesting, like in the duodenum, a post-transcriptional regulation. In the spleen, iron is detected mostly in macrophages expressing the F4/80 antigen [38]. To ensure that the low level of iron in the spleen of the CFTR KO mice was not due to the lack of macrophages, q-PCR and F4/80 immunostaining were performed that allow to detect similar F4/80 mRNA levels and positive cells in both mutated and WT mice (S3 Fig). Noteworthy, no signs of erythroid hyperplasia that could explain the iron deficiency of splenic macrophages were detected. Indeed, CFTR KO mice display no splenomegaly, and the mRNA levels encoding the heme biosynthetic enzymes eALAS and ferrochelatase were normal (S3 Fig).

The iron deficiency of splenic CFTR KO macrophages could be explained either by increased iron export and/or decreased iron acquisition. Overexpression of ferroportin could explain the iron deficient status of the splenic CFTR KO macrophages. However, in the spleen, we detected no change of ferroportin protein levels (Fig 4C, bottom panel), thus favoring the second hypothesis, i.e. the splenic iron deficiency being caused by reduced iron acquisition. A possible reason for the iron deficiency of macrophages could be an impairment of the erythrophagocytosis (EP) process, EP being the predominant source of iron for the macrophages [39].

### Role of CFTR in heme export from the phagolysosome to the cytosol

Senescent RBCs internalized by phagocytosis are degraded within acidified early phagolysosomes. Heme is then transported into the cytosol by the membrane heme transporter HRG1 [40] and catabolized by HO-1 yielding to the release of iron. Iron in turn regulates the IRE-dependent gene expression, leading in particular to the up-regulation of ferritin synthesis allowing protection of the cell by chelation of the pro-oxidant iron released from heme [23].

Interestingly, apart form its presence on epithelial cells, CFTR was reported to be also present at the membrane of organelles such as the endosome/lysososme and to cooperate with the vacuolar proton ATPase to acidify the lysosomes [41]. Given that the function of HRG-1 in promoting heme transport is dependent on V-ATPase activity, we hypothesized that CFTR protein could alter HRG-1-mediated heme export from the phagolysosome to the cytosol during EP.

**Fig 5:**
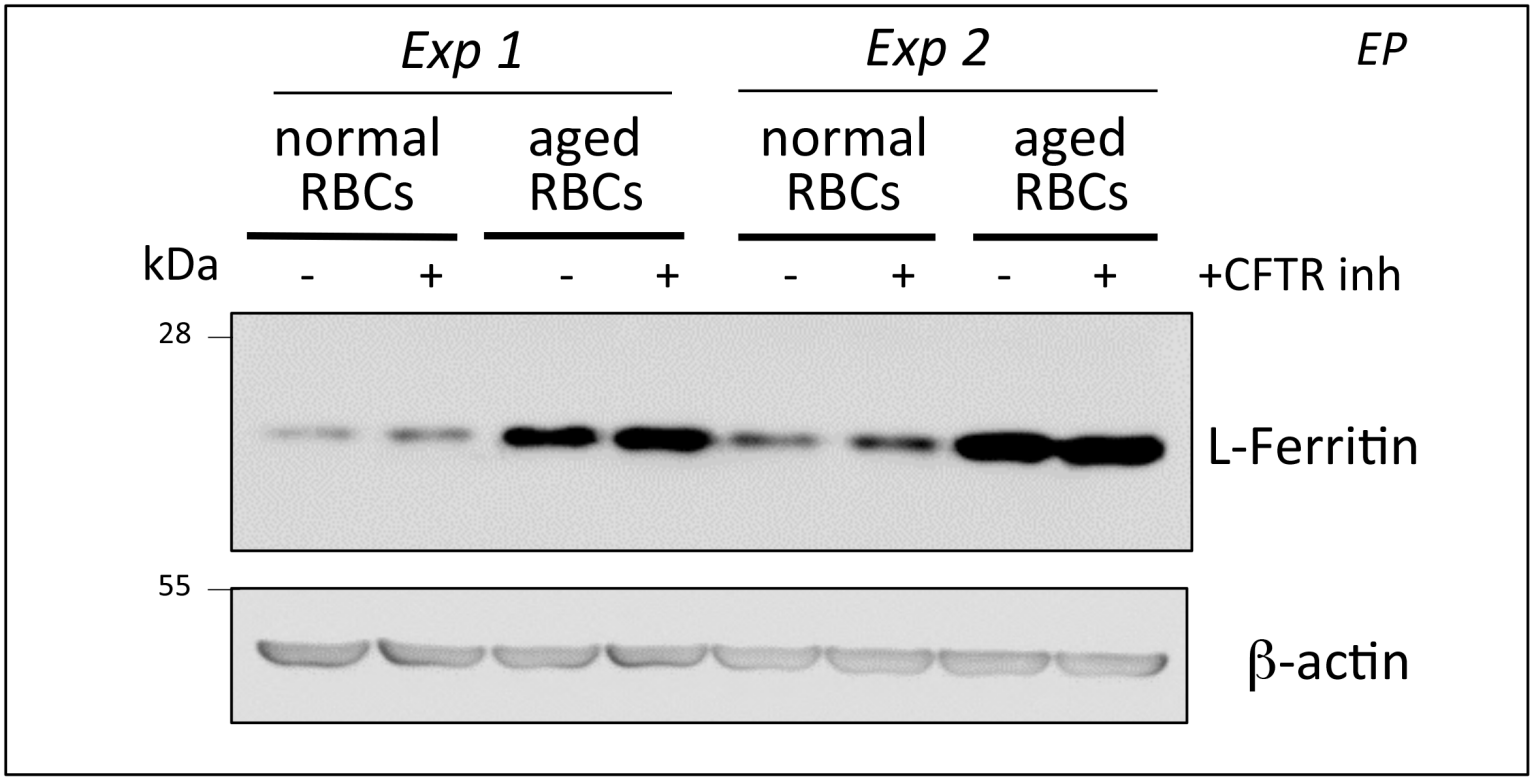
Erythrophagocytosis in presence of CFTR(inh)-172. Two independent experiments (Exp1, 2) of erythrophagocytosis (EP) using normal or aged red blood cells were performed with bone-marrow derived macrophages incubated or not with 10^-5^M CFTR(inh)-172.

To address this question, we used a well-characterized cellular model of EP and iron recycling by macrophages [23]. Primary culture of murine bone-marrow derived macrophages (BMDMs), were incubated with normal or artificially aged murine RBCs in presence or not of the extensively used CFTR inhibitor, CFTR(inh)-172 [42] at 10 μM. Six hours after EP, ferritin levels were measured by WB analysis to determine the efficacy of EP-mediated heme, and consequently, iron release, in the cytosol. As shown in Fig 5, EP-mediated ferritin up-regulation was unaffected by the presence of the inhibitor demonstrating that CFTR activity is most likely not required to ensure adequate ferritin synthesis after macrophage EP.

In conclusion, in this study, we demonstrated the absence of lung phenotype with regards to iron and inflammatory status in the CFTR KO mice and further reported a likely increased intestinal iron absorption rate allowing normal transferrin saturation levels. Nevertheless, CFTR KO mice presented with decreased plasma ferritin levels. Plasma ferritin being derived primarily from macrophages [43] and ferritin levels being unaltered in the liver of the CFTR KO mice, we propose that ferritin deficiency in the circulation may originate from spleen ferritin deficiency (See S4 Fig). So far, the molecular mechanisms of spleen ferritin deficiency are not clear. Ex vivo, we present evidence for unaltered ferritin release during the late steps of EP. Nevertheless, *in vivo*, CFTR being present on the RBC [44], the first steps of EP, i.e. recognition and binding of RBC to spleen macrophages, could be altered. Alternatively, the turn over of ferritin by the process of ferritinophagy could be modified in the absence of CFTR [45, 46]. Finally, we found that despite the systemic inflammatory status of the KO mice, liver hepcidin gene expression was not up-regulated but rather decreased. This decrease might be viewed as a consequence of the hypoferremia. However, whether decreased hepcidin could participate as a primary event responsible for increased iron absorption from the duodenum and iron efflux through ferroportin in the spleen remains a possibility that deserves further investigation (See S4 Fig).

Together, these results should provide a better understanding of iron dysregulation in CF patients where treating or not iron deficiency remains a challenging question, iron supplementation being associated with several theoretical concerns.

## Supporting Information

S1 Fig Iron-related phenoype of the liver in CFTR KO mice. Liver iron quantification (A), liver sections stained with Perl's Prussian blue (B) and liver L-ferritin analysis from cytosolic fractions, the right panel represents the quantification of the blots (arbitrary units) (C). Data are presented as mean ± SEM.
(TIF)

S2 Fig mRNA levels relative to cyclophilin-A expression were assessed by real-time PCR for the indicated genes in the duodenum (A and B) and the spleen (C). Data are presented as mean ± SEM. * P<0.05.
(TIF)

S3 Fig F4/80 immunohistochemistry on spleen section (A). Positively stained macrophage presenting a brown staining were present in both WT and KO mice. mRNA levels relative to cyclophilin-A expression were assessed by real-time PCR for the indicated genes in the spleen (B). Data are presented as mean ± SEM.
(TIF)

S4 Fig Model representation of iron distribution in CFTR KO mice. (TIF)
