# Supplementary figures and images for: Iron Homeostasis and Inflammatory Status in Mice Deficient for the Cystic Fibrosis Transmembrane Regulator
Source: PLoS One. 2015 Dec 28;10(12):e0145685. doi: 10.1371/journal.pone.0145685 (PMC4699203; doi:10.1371/journal.pone.0145685)

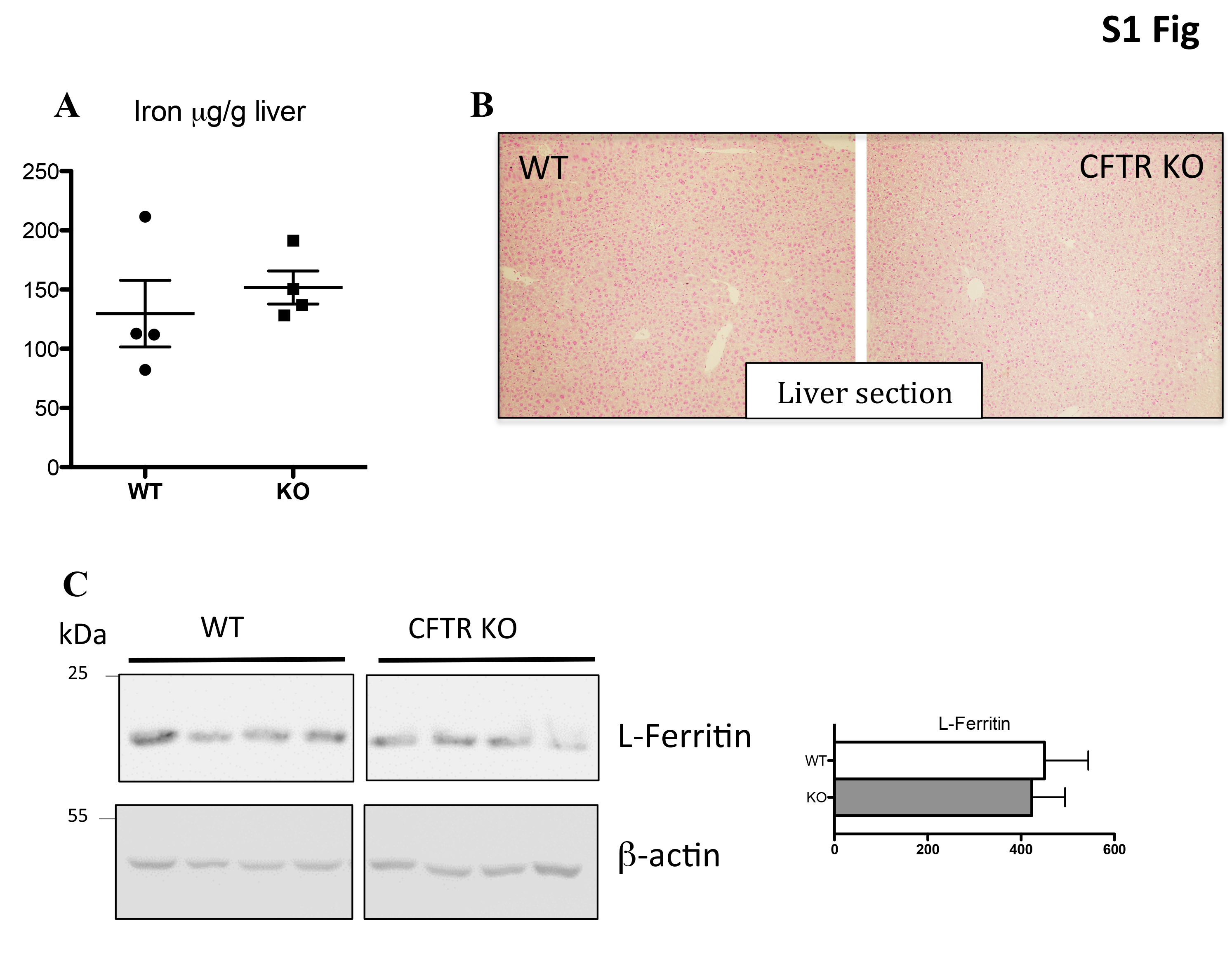

Supplement: S1 Fig — Liver iron quantification (A), liver sections stained with Perl's Prussian blue (B) and liver L-ferritin analysis from cytosolic fractions, the right panel represents the quantification of the blots (arbitrary units) (C). Data are presented as mean ± SEM. (TIF) [file pone.0145685.s001.tif]

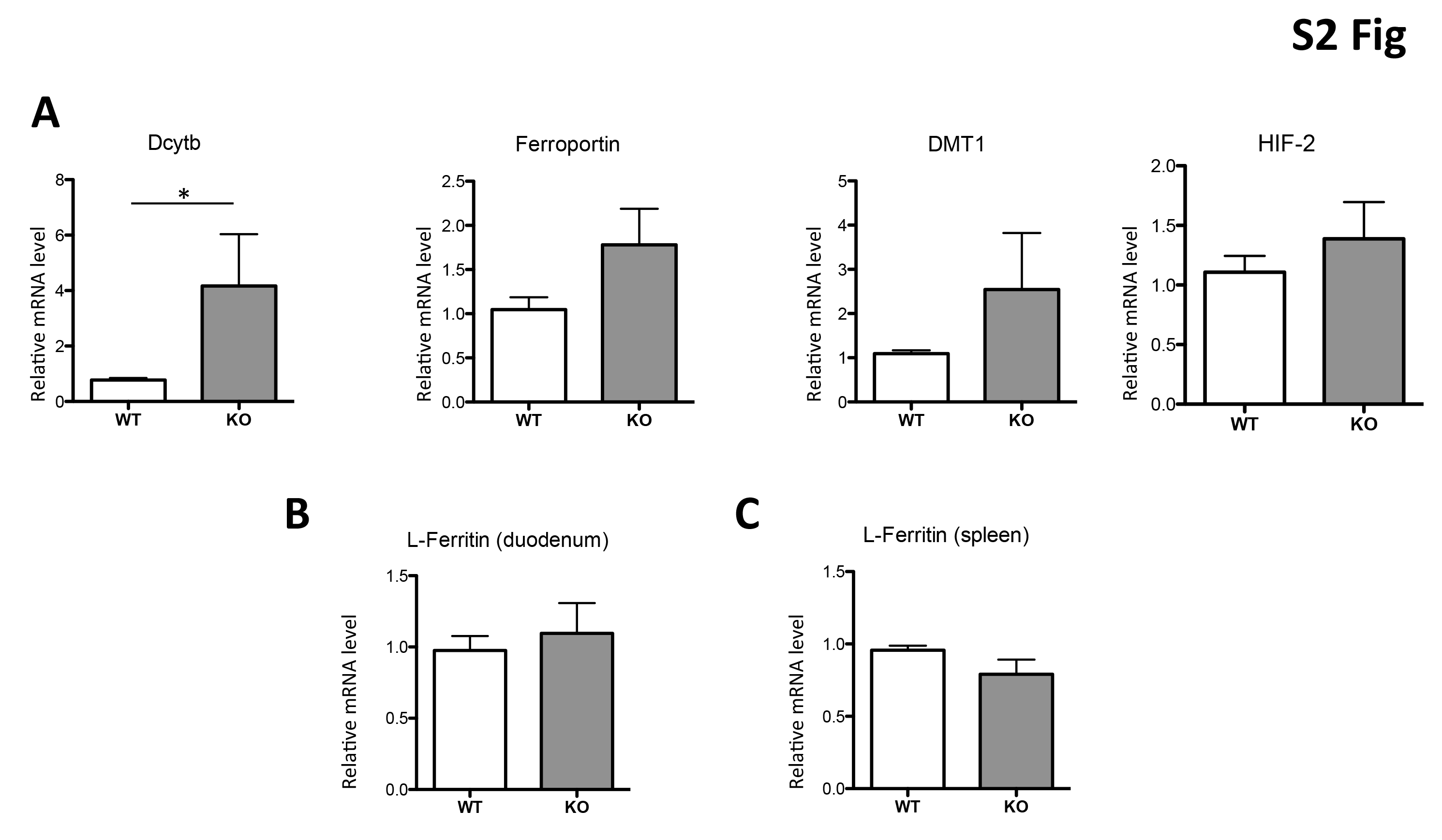

Supplement: S2 Fig — Data are presented as mean ± SEM. * P<0.05. (TIF) [file pone.0145685.s002.tif]

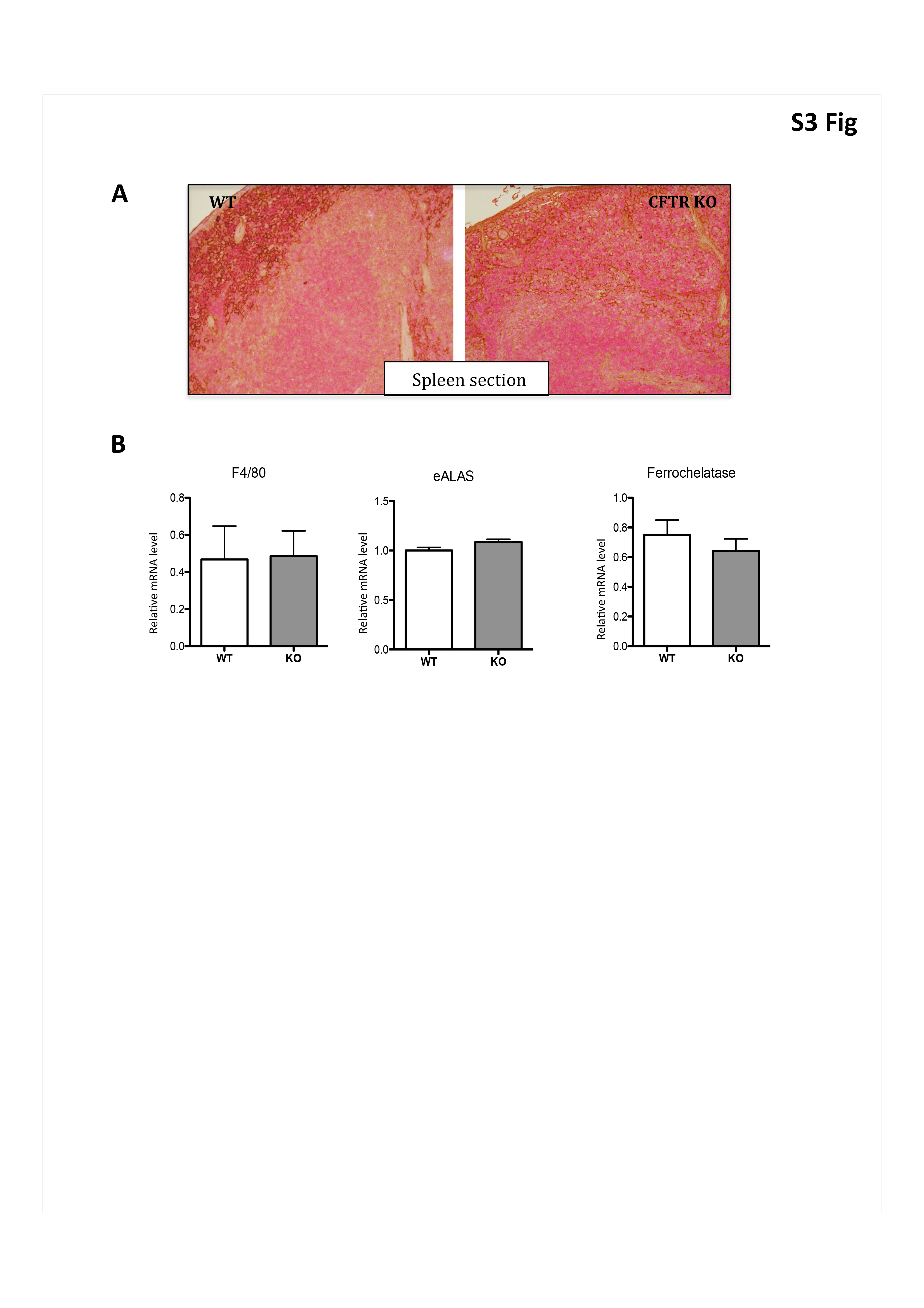

Supplement: S3 Fig — F4/80 immunohistochemistry on spleen section (A). Positively stained macrophage presenting a brown staining were present in both WT and KO mice. mRNA levels relative to cyclophilin-A expression were assessed by real-time PCR for the indicated genes in the spleen (B). Data are presented as mean ± SEM. (TIF) [file pone.0145685.s003.tif]

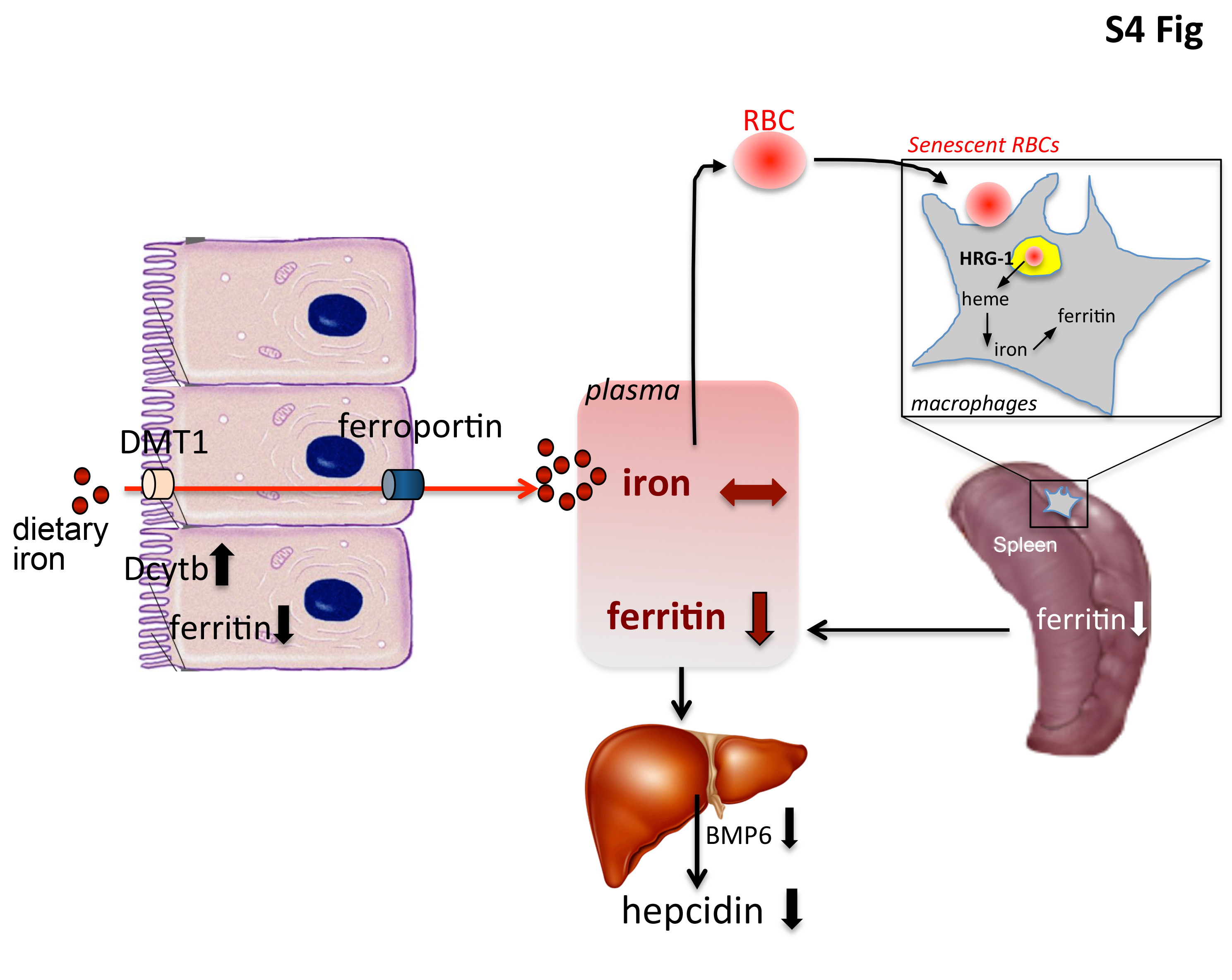

Supplement: S4 Fig — (TIF) [file pone.0145685.s004.tif]
